# The Hospital World

**Published:** 1910-04-16

**Authors:** 


### The Hospital World.

### Elections and Appointments.

The Lord Mayor, Sir J. Knill, has been unanimously elected president of the Council of the Hospital Saturday Fund.

Mr. D. D. Kirkaldy, the hon. secretary of the South Wimbledon and Merton Cottage Hospital, has been re-elected.

The Duke of Connaught has been elected President of the Hospital and Home for Incurable Children, North-court, Hampstead.

Dr. Crowe, Mr. T. Bates, jun., and Mr. Pollard have been appointed to act upon the executive committee of the Worcester General Infirmary.

The Following Gentlemen have been elected to the Executive Committee of the Worcester Infirmary: Dr. Bates, Father Kenny, the Rev. C. J. Hunt, Mr. C. Raymond, Mr. Bruce Ward, and Mr. F. Wood.

The Eastleigh Hospital Committee has chosen, on the motion of Mr. J. W. Angel (hon. treasurer), seconded by Mr. J. Treherne, Mr. Surrey Warner to be president, and Mr. E. Rowe to be chairman. Mr. J. W. Angel has been in turn unanimously re-elected treasurer, and Mr. F. J. Hendy hon. secretary. Mr. A. C. Crew has been appointed to the post of assistant secretary.

The Marquis of Hertford has been re-elected president of the Birmingham and Midland Ear and Throat Hospital; the Earl of Dudley, the Bishop of Birmingham, the Lord Mayor of Birmingham, Sir W. Foster, Alderman Beale, Alderman Clayton, Mr. W. B. Cregoe-Colmore, and Mr. J. G. Johnson, vice-presidents; and the following as members of the committee: Messrs. J. H. Barclay, T. W. Browett, C. S. Court, W. J. Gell, F. W. V. Mitchell, J. M. Smith, J. C. Vaudrey, and J. Whitfield.

The Board of Management of the Warrington Infirmary and Dispensary has been re-elected as follows: The president and vice-presidents, Mr. H. Thornton, Lieutenant-Colonel Buckton, Rev. Father Whittle, Rev. E. C. E. Carleton, Alderman Forshaw, Rev. W. Bracecamp, Councillor Jackson Holmes, Captain Crosfield, Rev. C. Harvey Cook, Mr. J. H. Smethurst, Councillor H. Roberts, Mr. F. W. Monks, Mr. J. A. Ransome, Alderman Burton, Mr. William Daintith, Mr. George Walker, and Mr. M. McMillan.

Sir Gilbert Greenall, Bart., has been re-elected president of the Warrington Infirmary and Dispensary. The following gentlemen are again vice-presidents: The Mayor of Warrington, Mr. T. H. Lyon, Mr. J. Charlton Parr, Rev. Canon Willis, Mr. F. Monks, Mr. Charles White, Mr. George Dakin, Mr. John Fairclough, Mr. Ziba Armitage, Mr. Roger Charlton Parr, Mr. A. H. Crosfield, M.P., Mr. W. Peter Rylands, Rev. John Yonge, Rev. Harry V. Pigot, Mr. William Long, Mr. Henry Greenall, Mr. W. H. Bleckly, Mr. J. J. Bleckly, and Mr. William Carson.

Mr. Sinden has been unanimously re-elected chairman, with Mr. W. P. Luxford as vice-chairman of the Forest Row and District Working Men's Hospital Committee. Mr. Sinden, moreover, has been re-appointed to represent the committee on the East Grinstead Cottage Hospital Committee. The Working Men's Hospital Committee of Forest Row consists of the following gentlemen: Mr. W. F. Grinham, Mr. T. Taylor, Mr. C. H. Hinkley, Mr. W. H. Godden, Mr. H. Wheatland, Mr. H. Card, Mr. W. Nice, Mr. H. Horsey, Mr. E. Hemsley, Mr. H. Welfare, Mr. W. Beedle, Mr. C. H. Lindfield, Mr. J. Hider, and Mr. H. Wyatt. The Committee's secretary is again Mr. Mills.

New members of the Council of the Chelsea Hospital for Women are the Hon. Edward Cadogan, Sir Samuel Provis, K.C.B., Dr. F. H. Anderson, Mr. Thomas J. Whiffen, and Mr. Herbert J. Mappin. Muriel Viscountess Helmsley, Mrs. im Thurn, and Mrs. Victor Bonney have joined the Ladies' Committee.

### Personalia.

The Committee of Management of the Morningfield Hospital, Aberdeen, has resolved to place on record its deep regret at the recent death of Colonel James Allardyce, LL.D., of Culquoich, who for nearly fourteen years had been a member of the Committee of Management, and had been a keen hospital worker to the last. The committee also directed an extract of this resolution to be sent to Miss Allardyce as an expression of the committee's sympathy with her and the other members of the deceased's family in their loss.

Fifteen Years Ago the Rev. W. H. Orton took up the secretaryship of the Sussex County Hospital. During that period four chairmen in turn have vied with one another in praising him for his work, and in pointing out to a sometimes dull public how much it has owed to his industry, his personal kindness, and his ability. When the public feels that it is receiving good returns for the money it subscribes to a hospital, wise men know that the Secretary is doing good work. The reason for Mr. Orton's resignation we give in his own words: "I recognise that the time has come when for my own sake and the good of this Institution, which I have so much at heart, the severance must be made. My regret in taking this course is greatly mitigated by the knowledge that I shall, as Chaplain, have the privilege of a seat on the Board of Management, and so be enabled to enjoy the friendship and kindness which I have always experienced, and so greatly appreciated, and for which I most gratefully thank you." It should be added that Mr. Orton's resignation will not take effect till June 24.

A Line of Hospital Presidents makes often very good history when traced out. Here is an example: In supporting the election of Mr. J. Herbert Benyon to the office of president of the Royal Berkshire Hospital, Mr. F. C. C. Barnett brought to light the following facts, which are worthy of record. "I do not think," Mr. Barnett remarked, "that the close connection of the name of Benyon with this hospital in its early days is generally known and appreciated. The hospital would never have been built if Mr. Richard Benyon de Beauvoir had not subscribed the first £5,000. He became the first president of the hospital, the first report of which was issued almost exactly seventy years ago. During those seventy years there have been only four presidents of the hospital. Mr. Richard Benyon de Beauvoir, who occupied the office of president for ten or eleven years, was succeeded on his death by Mr. Richard Benyon. Those two gentlemen held the reins of office for no fewer than fifty-seven years. They were noted throughout the county for their philanthropy, liberality, and large-mindedness, and saw, what many did not see at that time, the possibility of the growth of that hospital. I do not think it is possible to imagine that under a broken series of presidents the hospital would have reached its present position. It seems most appropriate that the chief office in the first charitable institution of the county should be carried on again by a member of the family, and I look forward to Mr. Herbert Benyon bringing with him the great traditions of his family."

